# Case Report: Peripheral combined central dual-target magnetic stimulation for non- motor symptoms of Parkinson’s disease

**DOI:** 10.3389/fpsyt.2025.1556045

**Published:** 2025-04-25

**Authors:** Shangxiaoyue Li, Hongwei Cai, Xiaoyu Liao, Aihong Li, Xiaosu Gu, Aisong Guo

**Affiliations:** ^1^ Rehabilitation Medical Center, Affiliated Hospital of Nantong University, Nantong, China; ^2^ School of Nursing and Rehabilitation, Nantong University, Nantong, China; ^3^ Department of Neurology, Affiliated Hospital of Nantong University, Nantong, China

**Keywords:** Parkinson’s disease, vagus nerve, repetitive transcranial magnetic stimulation, non-motor symptoms, electroencephalogram

## Abstract

This case report describes an innovative study using central combined vagus dual-target magnetic stimulation for treating non-motor symptoms of Parkinson’s disease (PD). PD is a common neurodegenerative disease, and almost all PD patients experience varying degrees of non-motor symptoms. However, there aren’t many targeted drugs for non-motor symptoms. Based on this clinical, we used left dorsolateral prefrontal cortex (DLPFC) and vagus nerve dual-target magnetic stimulation to treat PD non-motor symptoms. The choice of this combined stimulation method is based on the closed-loop rehabilitation theory of central-peripheral-central. Stimulation of DLPFC promoted the activation of brain functional areas and improved neuroplasticity, while stimulation of vagus nerve further enhanced the positive feedback and input to the central nervous system, forming a closed-loop information feedback, and synergically promoted the recovery of PD non-motor symptoms. The patient in this paper had non-motor symptoms such as constipation, short-term memory impairment, insomnia, depression, hallucinations. We had 10 sessions in total. The DLPFC stimulation was performed at 10Hz, 120% resting motor threshold (RMT) intensity, 1000 pulses per sequence for 10 minutes. The vagus nerve stimulation was performed at 10Hz, 100%RMT, with a total of 2000 pulses and a duration of 14 minutes. Assessment before treatment, after treatment, and at one month follow-up showed improvements in cognitive function, mood, and constipation symptoms. Therefore, we believe this treatment approach may represent a promising new option for treating non-motor symptoms of PD.

## 1 Introduction

PD is among the most rapidly increasing neurological disorders globally in terms of incidence (1). With the degeneration of dopaminergic neurons in the substantia nigra pars reticulata and the inner segments of the pallidum, it leads to the gradual emergence of both motor and non-motor symptoms in PD patients (2). In addition to common motor symptoms like tremor, stiffness, and postural instability, non-motor symptoms such as depression, sleep disorders, cognitive impairment, and autonomic dysfunction significantly impact the quality of life of PD patients (3). Although pharmacological interventions, such as dopaminergic medications, can improve symptoms in certain cases, they typically target early motor symptoms (4). It is therefore evident that non-motor symptoms also require non-pharmacological assisted treatments. Repetitive transcranial magnetic stimulation (rTMS) has emerged as a promising non-invasive neuromodulation technique for PD (5), capable of improving non-motor symptoms (6, 7). By delivering repeated magnetic pulses to specific brain regions, rTMS regulates cortical excitability and modulates connected brain networks, thereby restoring dysfunctional neural circuits (8). Regarding target selection, stimulation of DLPFC can regulate neurotransmitters and improve cortical plasticity (9, 10). It plays a key role in executive function, working memory and emotional regulation (2, 11). At the same time, vagus nerve stimulation can directly affect autonomic nerve function (12), and connect the central nervous system through the gut-brain axis (GBA), which further enhances the positive feedback and input to the central nervous system to achieve the purpose of improving non-motor symptoms (13–15). Based on the central-peripheral-central closed-loop rehabilitation theory, combining these two targets creates a complete closed-loop system. This synergistic approach enhances neural circuit remodeling and promotes patient functional recovery (16). In addition, previous studies have found similar views. They used magnetic stimulation to intervene in the central and peripheral nerve target areas, and found that multi-target stimulation could better enhance the regulation of nerve circuits, rebuild the damaged nerve function, and promote the functional recovery of patients (17–19). In view of these findings, we believe that a single stimulus target may not be as effective as multi-target stimulation therapy (20–23). Therefore, our current study aims to explore the efficacy of dual-target magnetic stimulation in DLPFC and vagus nerve in improving the non-motor symptoms of PD.

## 2 Case description

The 73-year-old female patient was diagnosed with PD 8 years ago. Initially, she presented with bradykinesia in both lower limbs and tremors in the right upper limb; however, these symptoms were left untreated. Over the years, her condition progressed, leading to significant bradykinesia, hyposmia, and short-term memory impairment that began manifesting 4 years ago. Subsequently, she was prescribed a medication regimen that included Entacapone and Pramipexole, resulting in noticeable improvement. Hallucinations occurred 2 years ago, and the patient’s family noticed that she often talked to herself and had visual hallucinations. There was no fixed time when they occurred, and the frequency could reach 3-4 times a week. This led her to adjust her medication regimen with modest success. In the past 3 days, the patient’s conscious symptoms worsened again, resting tremors developed in both upper limbs and right lower limb, difficulty turning over at night, constipation, short-term memory impairment, depression and other symptoms appeared. The efficacy of her medication declined, providing relief that lasted only three hours. She was subsequently hospitalized. Main examination results: Head MRI showed increased iron deposition in the pallidum and putamen on both sides. (**Supplementary Figure 1**). Plasma phosphorylated Tau-181 and beta-amyloid (1-42) showed no significant abnormalities. She continued her medication regimen while in hospitalized: Levodopa and Benserazide Hydrochloride Tablets (1/4 tablet four times a day), Entacapone (1 tablet once daily), and clozapine (1/4 tablet twice a day). The type and dosage of drugs remained unchanged during hospitalization. The patient took the drugs alone, these non-motor symptoms did not improve, and she lost confidence in the efficacy medication. Given these circumstances, the patient participated in our clinical trial of central combined with vagus nerve dual-target magnetic stimulation therapy. **Table 1** shows the baseline clinical characteristics of this patient.

**Table 1:** Characteristics of PD case.

Characteristics	Describe
Age (y)	73
Education (y)	6
Sex	female
Disease duration (y)	8
Hoehn and Yahr (stage)	3
Tremor type	Resting tremor
UPDRS-III	34

UPDRS-III, Unified Parkinson’s Disease Rating Scale-Part III.

## 3 Materials and methods

The patient received conventional levodopa-based medication at the Department of Neurology and received education from the Department of Rehabilitation staff. We use the YRD CCY-1 transcranial magnetic therapy device manufactured by Wuhan Yiruide Medical Equipment Company. The diameter of the circular coil is 12.5cm. The patient is fitted with a TMS positioning cap that fits the head shape and adjusts the position according to the occipital nodule. The patient is placed in a supine position and the coil center is aligned to the left DLPFC region on the TMS positioning cap. The stimulation parameters were: frequency 10 Hz and an intensity of 120% RMT, with 1000 pulses per sequence over 10 minutes. Subsequently, the patient was placed in the right lateral position, with the central point of the circular coil placed on the vagus nerve (left mastoid) (14), delivering stimulation at 100% RMT intensity, 10 Hz frequency, 50 pulses, 16-second intervals, and a total of 2,000 pulses, for a duration of 14 minutes. During the treatment, we hold the coil throughout to eliminate the possibility of deviation from the treatment target due to the patient’s movement. Treatment was administered once daily, five days per week (Monday to Friday), for two consecutive weeks, totaling 10 sessions. We assessed before and after treatment, and one month after treatment. The flow chart is shown in **Supplementary Figure 2**. The evaluation used the following indicators: (1) Montreal Cognitive Assessment (MoCA) to assess patients’ cognitive function. (2) Hamilton Depression Scale (HAMD-24) assessed depressive symptoms. (3) Hamilton Anxiety Scale (HAMA) to assess patients’ anxiety symptoms. (4) Scales for Outcomes in Parkinson’s disease Autonomous (SCOPA-AUT) assessment of patients’ autonomic nervous system function. (5) Non-Motor Symptoms Scale for Parkinson’s Disease (NMSS) assesses the frequency and degree of non-motor symptoms in patients. (6) Parkinson’s Disease Sleep Scale (PDSS) assessed patients’ sleep status. (7) Parkinson’s Disease Questionnaire (PDQ-39) assessed patients’ quality of life. (8) Electrophysiological measures: resting state EEG, event related potential (ERP) P300, and RMT. The study was conducted in accordance with the Declaration of Helsinki, and approved by the Ethics Committee of the Affiliated Hospital of Nantong University (protocol code 2024-K049-01). Written informed consent has been obtained from the patient to publish this paper and identifiable data were anonymized. This study has been registered in the U.S. Clinical Trials database (registration number NCT06009471).

### 3.1 Resting motor threshold

Prior to the TMS treatment, the RMT was assessed. The RMT of the abductor pollicis brevis (APB) muscle was evaluated using the electrophysiological assessment module of the YRD CCY-I TMS device. Prior to the evaluation, the patient was briefed on the procedure and precautions, and the patient’s consent was obtained. The patient is fitted with a TMS positioning cap that fits the head shape and adjusts the position according to the occipital nodule. Subsequently, the patient lies flat on a comfortable bed with palms facing upwards and body relaxed. We cleaned the right APB muscle of the patient with 75% alcohol, and the recording electrode was attached to the muscle belly. The midpoint of the circular coil was aligned to the left primary motor cortex (M1) on the TMS localization cap. A single pulse was triggered at 30% of the maximum output intensity, then the stimulation intensity was gradually increased until at least 5 out of 10 consecutive times were able to induce a motor evoked potential with an amplitude greater than 50μV. The minimum stimulation intensity at this time was recorded as the patient’s RMT (24). According to the position relationship between M1 and DLPFC and the TMS positioning cap, we determined the specific location of DLPFC and adjusted the stimulus intensity according to RMT.

### 3.2 EEG resting state

The 5-minute resting state EEG was evaluated using the NSM2 32-channel EEG detection system. During the collection of EEG signals, the patient was comfortably seated in a chair, instructed to remain relaxed, awake, and with eyes closed. Electrodes were positioned on the scalp based on the international 10-20 system standard, with reference electrodes (A1, A2) situated on the earlobes bilaterally. Electrode locations are shown in **Supplementary Figure 3**. We used MATLAB software for initial pre-processing of the EEG data, followed by fast Fourier transform (FFT) analysis to assess the patient’s average power spectral density (PSD) and power in the delta frequency band.

### 3.3 EEG oddball mode

The assessment was conducted using the NSM2 medical ERP system in a quiet environment, with the patient seated and attentive. We explain the purpose of the assessment to the patient, require general relaxation and concentration, and begin the formal test after the patient has fully mastered the assessment requirements. The entire test took about 7 minutes. Using the auditory Oddball paradigm, the recording electrode was the Cz site (with a total of 32 channels), with reference electrodes placed on the earlobes (A1, A2). The patient wore headphones and received both standard and target stimuli, with the target stimulus appearing in the standard stimuli with a 20 percent probability. The positive potential observed between 250-500 ms post-stimulus onset was known as the P300 (25). We mainly analyzed the latency and amplitude of the P300.

## 4 Results

After treatment, the patient’s memory impairment, anxiety, and depression symptoms improved significantly. These benefits were sustained at the one-month follow-up. However, no significant improvement in sleep quality was observed.

### 4.1 Scale score

The MoCA score improved from 16 points pre-treatment to 20 points post-treatment, and remained at 20 points at the one-month follow-up, suggesting enhanced cognitive function, particularly in short-term memory, with sustained improvement. Emotional state also showed marked improvement, with the HAMD-24 score decreasing from 14 to 10 points and the HAMA score dropping from 16 to 10 points, both of which remained stable at follow-up, indicating sustained relief from depression and anxiety symptoms. The SCOPA-AUT score dropped from 21 to 13, reflecting significant relief of autonomic symptoms such as constipation. The NMSS score decreased from 49 to 29 after treatment and further decreased to 26 at follow-up. The PDQ-39 score also improved, dropping from 53 to 40 at follow-up. However, the PDSS score showed minimal change, increasing only slightly from 104 to 107 points, indicating no significant improvement in sleep quality. The changes in each scale before and after treatment, as well as at follow-up, are detailed in **Table 2**.

**Table 2:** Changes of scale scores and RMT before and after treatment and at follow-up.

Scale	Pre-treatment	Post-treatment	Follow-up
NMSS	49	29	26
MoCA	16	20	20
PDSS	104	106	107
HAMA	16	10	10
HAMD-24	14	10	10
SCOPA-AUT	21	14	13
PDQ-39	53	44	40
RMT (%)	35	35	35

NMSS, Non-Motor Symptoms Scale for PD; MoCA, Montreal Cognitive Assessment; PDSS, PD Sleep Scale; HAMA, Hamilton Anxiety Scale; HAMD-24, Hamilton Depression Scale; SCOPA-AUT, Scales for Outcomes in PD Autonomic; PDQ-39, the 39-item PD Questionnaire; RMT, resting motor threshold.

### 4.2 Electrophysiological results

The changes of RMT before and after treatment are shown in **Table 2**. There was no significant difference before and after treatment, remaining at 35%.

**Figure 1:**
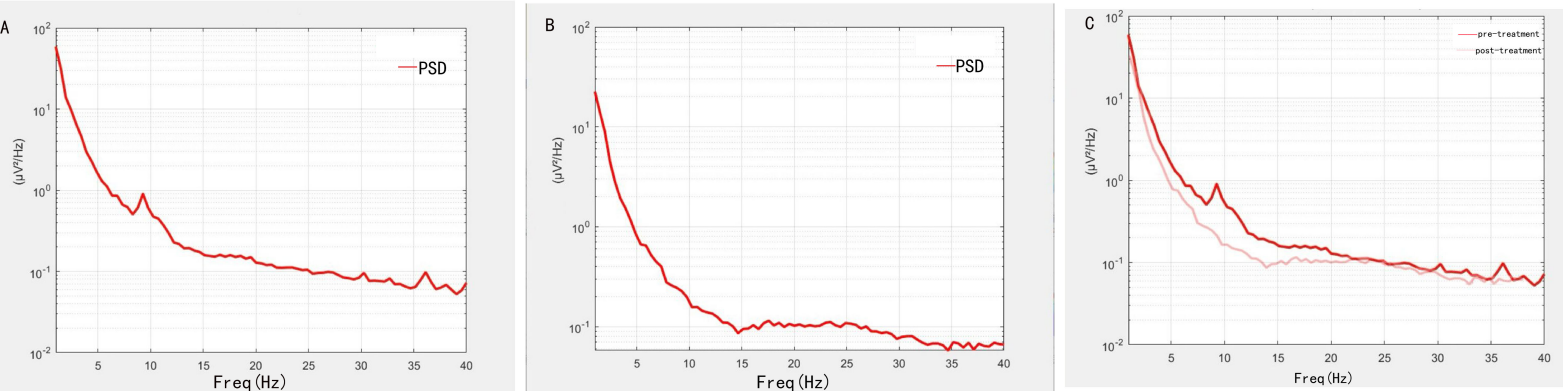
Changes in PSD shape before and after treatment. **(A)** The shape of PSD before treatment. **(B)** The shape of PSD after treatment. **(C)** The difference of PSD shape before and after treatment. Dark colored lines indicate before treatment, and light colored lines indicate after treatment.

Before treatment, the shape of power spectral density (PSD) showed significant fluctuation around 10Hz (**Figure 1A**), which was inconsistent with the shape of normal PSD. However, the shape of the PSD gradually improved after treatment (**Figure 1B**). The changes in PSD shape before and after treatment are shown in **Figure 1C**.

**Figure 2:**
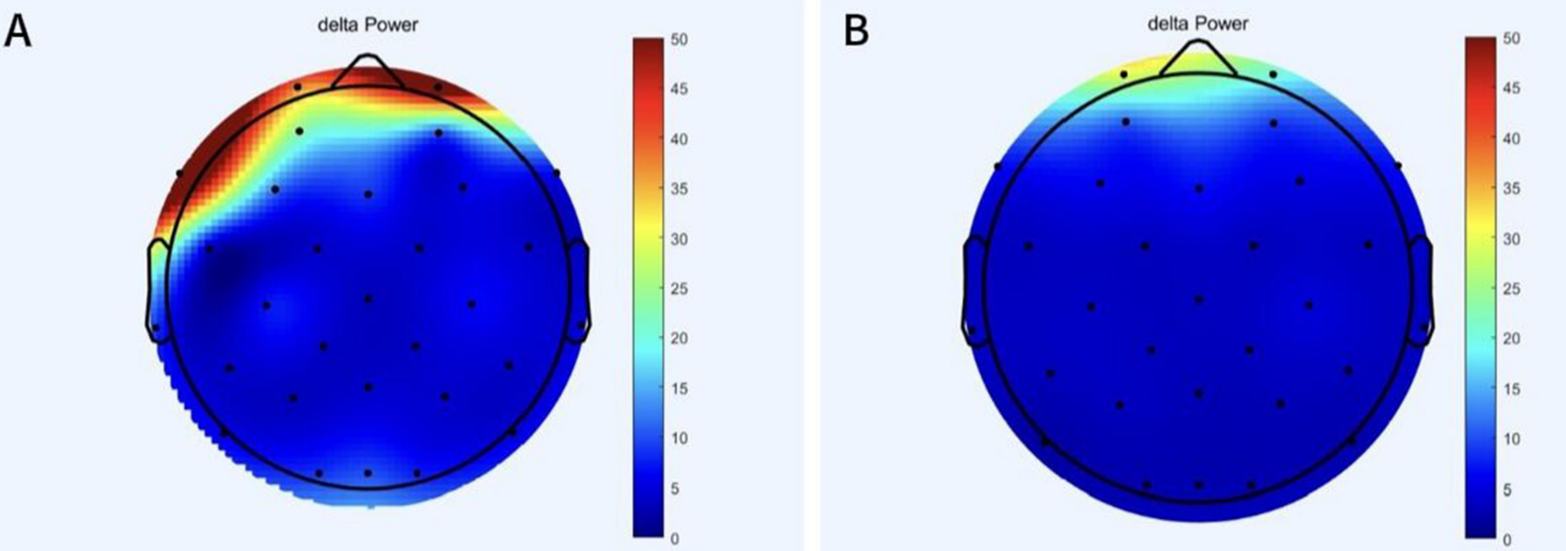
The change of delta band power before and after treatment. **(A)** Power in delta band of patient before treatment. **(B)** Power of delta band in patient after treatment.

The power of the patient’s delta frequency band before and after treatment is shown in **Figure 2**. Decreased delta power was observed after treatment compared to pre-treatment levels, especially in the frontal region.

The changes in P300 before and after treatment were observed through **Supplementary Figure 4** and **Supplementary Table 1**. The shape change in amplitude can be observed in **Supplementary Figure 4**, which shows different values as the amplitude changes over time. After treatment, the time corresponding to the peak of the maximum positive wave was shortened and the amplitude increased. Detailed numerical changes in amplitude and latency are provided in **Supplementary Table 1**, showing a decrease in latency from 352ms to 312ms and an increase in amplitude from 4.1μV to 4.3μV.

## 5 Discussion

To our knowledge, there has been no prior research of central rTMS combined with vagus magnetic stimulation for the treatment of non-motor symptoms of PD. In this case, our patient showed significant clinical improvement after treatment with no serious adverse effects observed. This suggests that 10 sessions of rTMS on the left DLPFC and vagus nerve may be able to improve the non-motor symptoms of PD.

After treatment, the patient’s cognitive function improved. We used MoCA and EEG results to assess cognitive function. The patient exhibited short-term memory impaired, resulting in a pre-treatment MoCA score of 16. However, after treatment, the MoCA score increased to 20 and remained stable at the one-month follow-up. This showed that the patient’s memory improved significantly after treatment, with the potential for long-term effects. Resting-state EEG data provided further objective evidence of these improvements. Specifically, before treatment, the shape of PSD in patients showed obvious fluctuation at about 10Hz, but after treatment, it tended to be normal. Power in the delta band also decreased significantly after treatment, especially in the frontal region. In addition, the P300 latency decreased from 352ms to 312ms, and the amplitude increased from 4.1μV to 4.3μV. The changes in these indicators are similar to those found in previous studies (26–30). Based on the above results, it is reasonable to speculate that the neuromodulation effects of treatment will persist in the short term as symptoms continue to improve. The possible mechanism for this result is that high frequency stimulation enhances cortical excitability (31, 32). Stimulation of DLPFC enhances afferent projection of caudate nucleus and putamen nucleus, affects long-term potentiation (LTP), enhances cortical plasticity and functional connection between brain regions (10, 33). It also synthesizes brain derived neurotrophic factor (BDNF), which promotes synaptic growth (34), allowing memory function to be restored. Stimulation of the vagus nerve can connect to the central nervous system via the GBA, further enhancing the positive feedback and input to the central nervous system (13, 35, 36). Through the closed-loop rehabilitation theory, peripheral intervention and central intervention can be organically combined to form bidirectional transmission and promote the establishment of functional synapses, thus leading to the improvement of cognitive function (16, 37). Previous studies have found similar findings. Sanders TH et al. reported that vagus nerve stimulation was associated with improved learning and memory (38). Trung J et al. found that after iTBS on the left DLPFC, the overall cognitive ability improved within a month, particularly in the areas of attentional and visuospatial (39). There have also been studies applying rTMS to two different targets, DLPFC and lateral parietal cortex (LPC), to observe the effects on cognition (40). Additionally, some meta-analyses have suggested that multi-target stimulation may be more effective in improving cognitive function (20).

The patient also showed marked improvements in emotional state, as indicated by reductions in HAMD-24 and HAMA scores from baseline to post-treatment. These changes are further supported by a decrease in frontal lobe delta band power. This is similar to previous research findings (41–44). Parkinson’s disease patients with comorbid depression often exhibit an imbalance of neurotransmitters in the brain (45). High frequency stimulation of the central nervous system can promote the release of dopamine in the striatum and regulate neurotransmitters in the brain, such as norepinephrine (9, 46). At the same time, vagus nerve stimulation has been shown to increase norepinephrine levels in the cortex, hippocampus, and medial prefrontal cortex, and regulate the imbalance of neurotransmitters in the brain. (47). Both can improve the neurotransmitter imbalance caused by anxiety and depression. In addition, meta-analysis suggested that multi-target combined stimulation could also improve depressive symptoms (22).

In addition to cognitive and emotional improvements, the patient exhibited alleviation of autonomic dysfunction, particularly constipation. The SCOPA-AUT score decreased from 21 to 13, reflecting improved bowel movement frequency and reduced symptom severity. This improvement may be attributed to the direct effect of vagus nerve stimulation on parasympathetic nerve activity (12), as well as improved autonomic nervous function through central and peripheral synergies (48). In addition, high frequency rTMS can enhance cortical excitability and release various neurotransmitters such as 5-hydroxytryptamine to regulate the control of the autonomic nervous system, so as to improve autonomic nervous function (49, 50). This is similar to previous research findings (51–53).

However, the PDSS scores remained unchanged, potentially due to the use of high-frequency rTMS, which may exacerbate hyperarousal in insomnia (31, 54). Future studies could explore low-frequency stimulation to address sleep disturbances more effectively.

Although the patient took medication during the hospitalization, we only carried out the central combined vagus dual-target magnetic therapy after it was clear that the drug treatment did not produce the desired effect, and the patient’s mood was increasingly anxious. The dosage and type of medication remained consistent throughout the treatment period and 1 month after treatment. However, it was only following our intervention that the patient’s non-motor symptoms improved. Furthermore, these symptoms remained under control during follow-up. Therefore, we attribute the improvement to the independent effects of our intervention, rather than the medication.

The positive outcomes we achieved are attributable to our treatment regimen and carefully optimized stimulation parameters. These parameters were developed based on previous research (9, 14, 55), patient feedback, treatment equipment specifications, and the central-peripheral-central closed-loop rehabilitation theory (16). This allows our stimulation regimen to maximize the impact on the cerebral cortex, promote neuroplasticity, and enhance the therapeutic effect. Despite the significant clinical results of our intervention, research on the optimal stimulation protocol for central and vagus nerve magnetic stimulation remains in its exploratory phase. In future studies, we aim to design controlled experiments varying stimulus intensity, duration, and the sequence of dual-target stimulation to identify the most effective treatment protocol.

Despite the promising results observed in this case report, several limitations should be acknowledged. First, the study was based on a single case with a small sample size. Second, the lack of a control group may limit the generalizability of the findings. But in the future, we will conduct larger sample sizes and randomized controlled designs to validate these preliminary findings. We hope that our study will provide valuable preliminary data for the design of future large sample randomized controlled trials.

## 6 Conclusions

In this report, we innovatively used central combined with peripheral dual-target magnetic stimulation for the first time in the treatment of PD and achieved good results. This approach effectively alleviated symptoms such as short-term memory impairment, depression, and constipation, showing potential for long-term efficacy. In the future, we will conduct randomized, double-blind and placebo-controlled trials with large samples.

## Data Availability

The original contributions presented in the study are included in the article/**Supplementary Material**. Further inquiries can be directed to the corresponding author/s.
