# Supplementary material for: Case Report: Peripheral combined central dual-target magnetic stimulation for non- motor symptoms of Parkinson’s disease
Source: Front Psychiatry. 2025 Apr 25;16:1556045. doi: 10.3389/fpsyt.2025.1556045 (PMC12062057; doi:10.3389/fpsyt.2025.1556045)
Supplement: Supplementary file 1 [file SupplementaryFile1.docx]

Supplementary Material


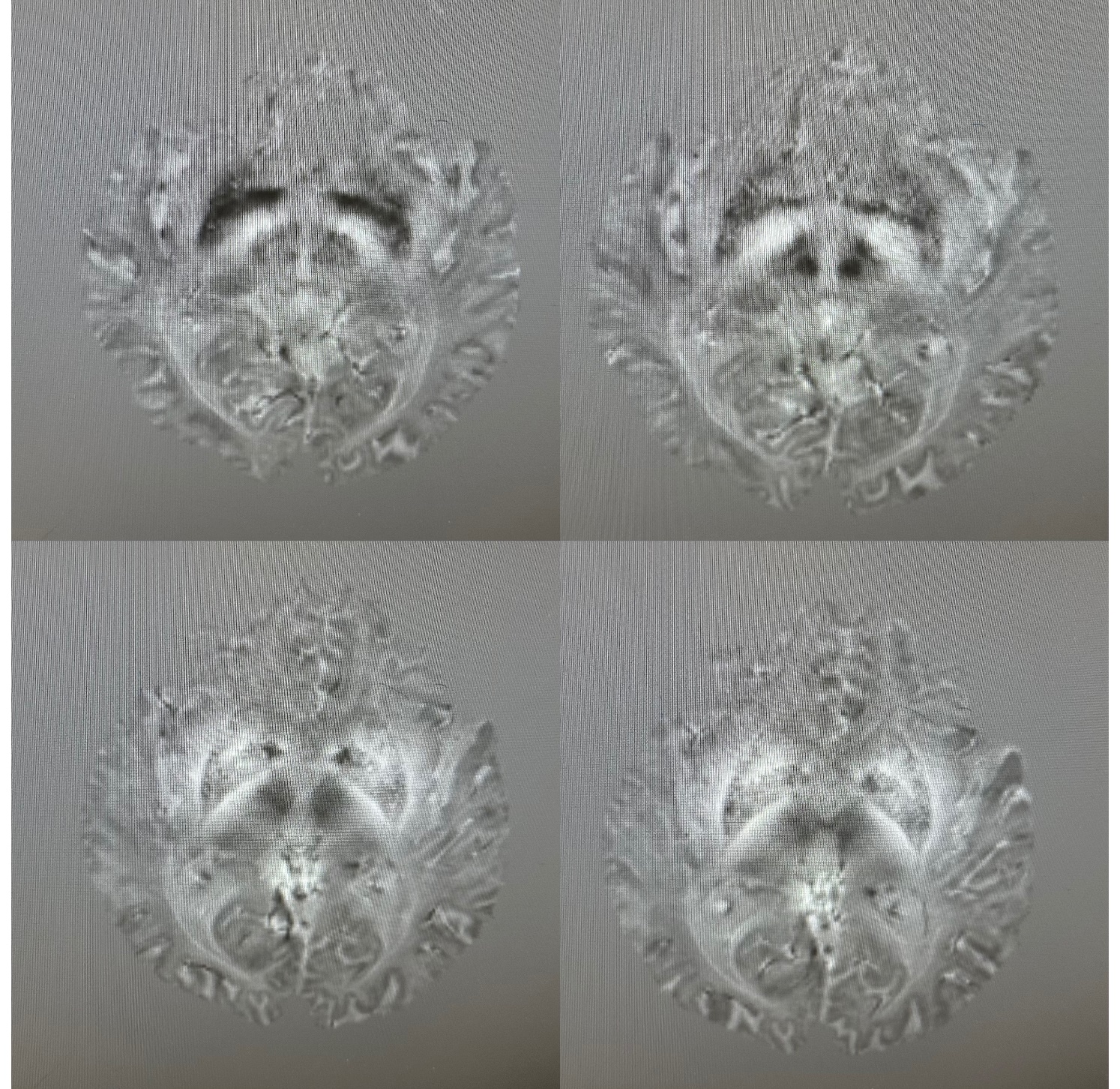


**Supplementary Figure 1.** MRI images of increased iron deposits in the brain of patients at specific time points.


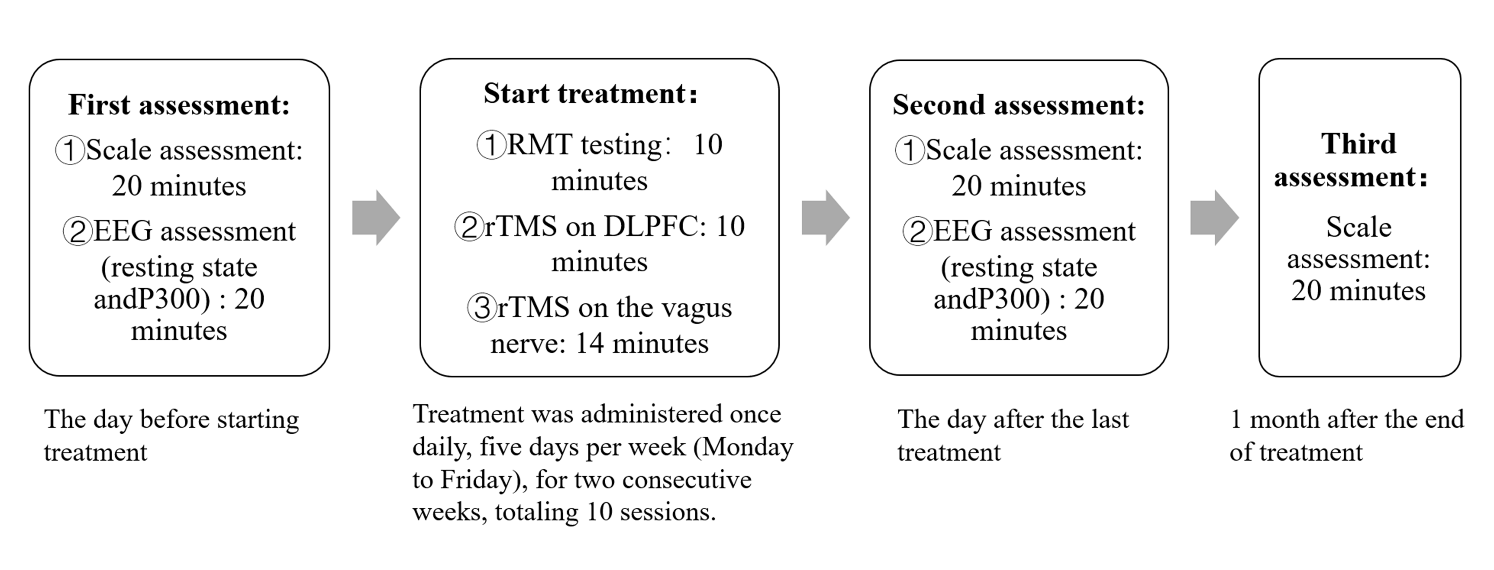


**Supplementary Figure 2. Flow chart**


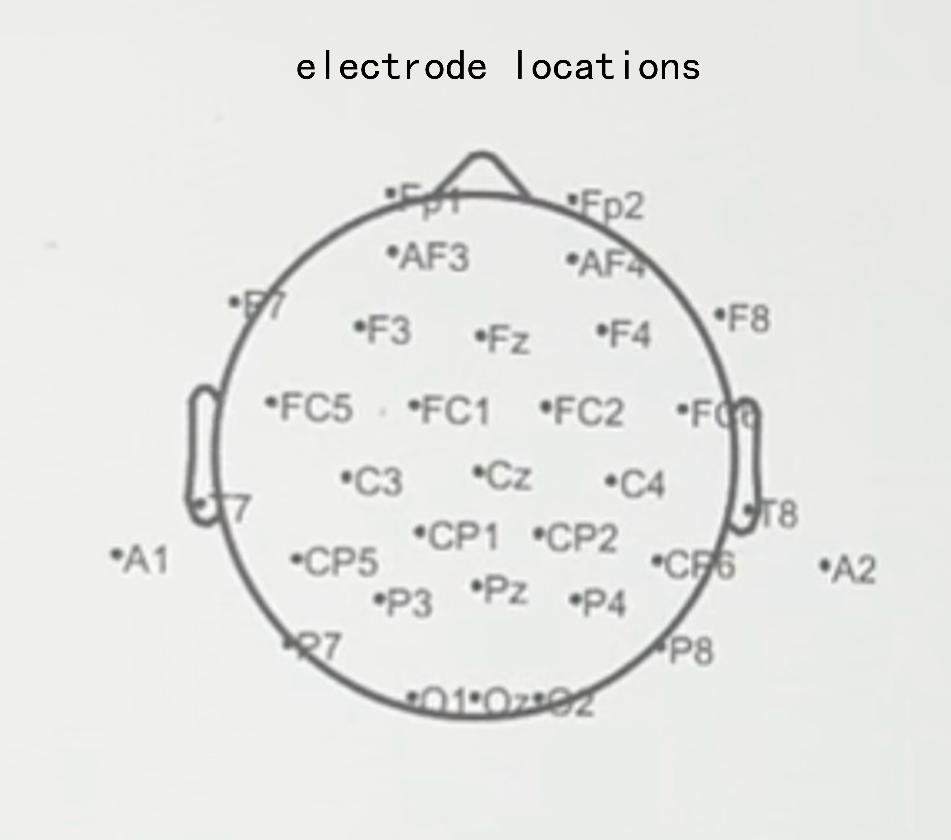


**Supplementary Figure 3. Electrode locations**

**
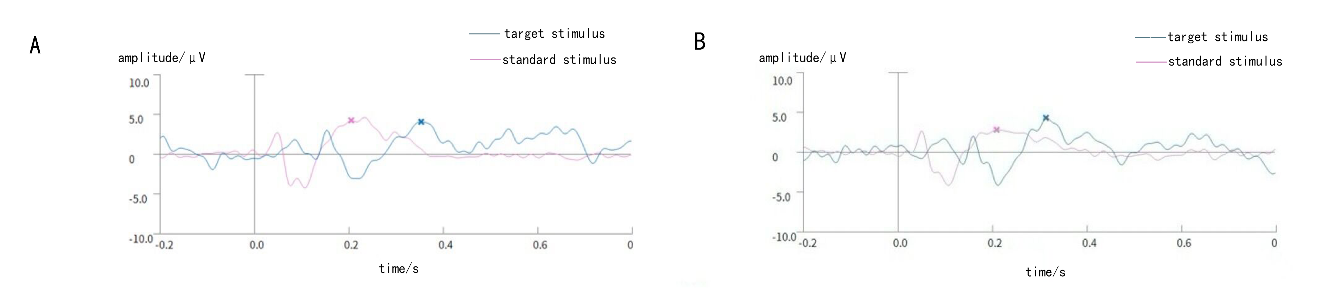
**

**Supplementary Figure 4.** Change in amplitude shape. (A) Change of P300 amplitude with time before treatment. (B) Change of P300 amplitude with time after treatment.

| Time | Latency | Amplitude |
| --- | --- | --- |
| pre-treatment | 352 ms | 4.1μV |
| post-treatment | 312 ms | 4.3μV |

**Supplementary Table 1:** Detailed numerical changes in amplitude and latency before and after treatment.
